# Understanding the Association Between Obesity and Obstructive Sleep Apnea Syndrome: A Case-Control Study

**DOI:** 10.7759/cureus.45843

**Published:** 2023-09-24

**Authors:** Kuldeep Patial, Hara Prasad Mishra, Giridhari Pal, Tarun Kumar Suvvari, Tamoghna Ghosh, Smruti Sikta Mishra, Chinmaya Mahapatra, Nidhal A Amanullah, Sara A Shukoor, Sibin Kamal, Indrajeet Singh, Juveriya Israr, Prem S Sharma, SN Gaur, Rajendra K Behera

**Affiliations:** 1 Sleep Medicine, Vallabhbhai Patel Chest Institute, University of Delhi, Delhi, IND; 2 Clinical Trial, All India Institute of Medical Sciences, New Delhi, Delhi, IND; 3 Pharmacology and Therapeutics, University College of Medical Sciences, University of Delhi, Delhi, IND; 4 Pharmacology, Vallabhbhai Patel Chest Institute, University of Delhi, Delhi, IND; 5 General Medicine, Rangaraya Medical College, Kakinada, IND; 6 Research, Squad Medicine and Research (SMR), Visakhapatnam, IND; 7 Medicine, All India Institute of Medical Sciences, New Delhi, Delhi, IND; 8 Occupational Therapy, Pandit Deendayal Upadhyaya National Institute for Persons with Physical Disabilities, New Delhi, IND; 9 School of Pharmacy, The Neotia University, Sarisha, IND; 10 Psychiatry and Behavioral Sciences, Sree Ramakrishna Mission Hospital, Thiruvananthapuram, IND; 11 Psychiatry, Government Medical College, Trivandrum, Trivandrum, IND; 12 Pain and Palliative Medicine, IQRAA International Hospital & Research Centre, Kandhla, IND; 13 Biotechnology, Rama University, Kanpur, IND; 14 Biosciences and Technology, Shri Ramswaroop Memorial University, Lucknow, IND; 15 Biostatistics & Medical Informatics, University College of Medical Sciences, Delhi, IND; 16 Pulmonary Medicine, Vallabhbhai Patel Chest Institute, University of Delhi, Delhi, IND; 17 Life Sciences, Sambalpur University, Sambalpur, IND

**Keywords:** case-control study, markers, bmi, anthropometric parameters, obesity, osa

## Abstract

Introduction

Obstructive sleep apnea (OSA) represents a sleep-related impairment linked to upper airway function. The question of whether OSA drives obesity or if shared underlying factors contribute to both conditions remains unresolved. Hence, this present study aims to understand the interplay between obstructive sleep apnea syndrome (OSAS) and obesity through in-depth analysis of anthropometric data within control subjects and OSA patients.

Methodology

A case-control study was conducted, which included 40 cases and 40 matched healthy controls. Study participants with reported symptoms of snoring, daytime drowsiness, or both were included in the study. All the study participants underwent comprehensive anthropometric assessments such as height, weight, body mass index (BMI), neck circumference, waist circumference, hip circumference, waist-to-hip ratio, skin-fold thickness, and thickness measurements of biceps, triceps, suprailiac, and subscapular muscles.

Results

Within the OSA group, significant disparities emerged in mean age, waist circumference, waist-to-hip ratio, and diverse fat accumulations encompassing visceral, subcutaneous, trunk, and subcutaneous leg fat. Notably, skin-fold thickness at specific sites - biceps, triceps, subscapula, and suprailiac - demonstrated considerable augmentation relative to the control group. Furthermore, mean values associated with height, weight, BMI, neck circumference, fat percentage, subcutaneous arm fat, entire arm composition, and trunk skeletal muscle either equaled or exceeded those in the control group. However, statistical significance was not attained in these comparisons.

Conclusion

This investigation underscored a pronounced correlation between numerous endpoints characterizing OSA patients and markers of obesity. Consequently, addressing altered levels of obesity-linked anthropometric variables through pharmacological interventions might hold promise as a pivotal strategy for improving symptoms associated with OSA.

## Introduction

Obstructive sleep apnea (OSA) is characterized by upper airway collapse during sleep, insufficient breathing, sporadic low oxygen levels, and sleep disturbance. OSA incidence and prevalence are influenced by age in the general population. The disease is more common in middle-aged and older people. If the apnea-hypopnea index (AHI) scores more than five, disease prevalence can reach up to 38%. This prevalence is higher among men than among women. In the general adult population, the occurrence of AHIs higher than 15% ranges from 6% to 17%, peaking at up to 49% in the senior citizen population [1].

Undiagnosed OSA has significant negative effects on the heart, metabolism, and brain, and it bears an increased risk of motor vehicle accidents and reduced quality of life [2,3]. Comorbidities are a leading area of study in OSA research. Recent studies have found that individuals with OSA have significantly higher frequencies of comorbidities than the general population [1-4]. When compared to individuals without OSA, males with OSA suffer from higher rates of diabetes and ischemic heart disease, while females experience higher rates of hypertension and depression [2,5].

Patients with OSA are commonly overweight or obese and experience drowsiness in sedentary situations or while driving. They also often have systemic hypertension, type 2 diabetes, and dyslipidemia [6]. Moreover, both obesity incidence and OSA diagnoses have been observed to increase. OSA prevalence can be as high as 40% in obese males with BMIs greater than 30 kg/m² [7] and can reach up to 90% for severely obese individuals with BMIs exceeding 40 kg/m² [8,9].

Previous studies have established a clear link between OSA and metabolic disorders. However, because of significant confounders like obesity, the role of OSA as an independent risk factor has become a subject of debate [10]. There are different views on the relationship between OSA and metabolic syndrome, with some suggesting that OSA is a separate risk factor for obesity [11]. OSA has been linked to hyperglycemia and dyslipidemia, even without any association with body fat [12]. It is uncertain whether obesity causes OSA, OSA causes obesity syndrome, or whether there are common factors that contribute to both conditions. Therefore, we conducted a case-control study to examine the anthropometric characteristics in control and OSAS patients.

## Materials and methods

A case-control study was conducted in the Department of Pulmonary Medicine, Vallabhbhai Patel Chest Institute, University of Delhi, Delhi, India, and the Department of Life Sciences, Sambalpur University, Odisha, India. Patients of the age group 30 to 60 years, exhibiting symptoms of either snoring, daytime drowsiness, or both and who provided consent were included in the study. Patients with preexisting medical conditions that could affect anthropometric measurements and individuals with a known history of sleep disorders other than OSA were excluded from the study.

Subject recruitment

For the study, adult males between the ages of 30 and 60 years who visited the hospital's outpatient department (OPD) complaining of snoring, daytime drowsiness, or both were included. Age-related sleep problems are more common in this age range. There were 40 patients in each of the two groups (control and OSA patients). Each subject received "written information" about the research on a "patient information sheet" after a detailed explanation and informed consent on a "patient consent form" was obtained before they were recruited in the study. The criteria utilized to evaluate individuals aged 30-60 years were obesity (BMI>30), ranging from >30 kg/m^2^ to 35 kg/m^2^; short neck; and history of snoring or excessive daytime sleepiness, fatigue, or insomnia.

Sample size

With one control for every instance, we conducted an independent case-control study. Previous research has found that the likelihood of exposure for controls is 13%. We must analyze at least 40 cases if the true odds ratio for sickness in exposed vs. unexposed individuals is 5, and control patients must reject the null hypothesis that this odds ratio is equal to 1 with a probability (power) of 0.8. The probability of committing a Type I error in this null hypothesis test is 0.05. We used either Fisher's exact test or the continuity-corrected chi-squared statistic to test this null hypothesis. Power and sample size calculation (**PS version 3.1.2) was used to compute the sample size.

Groups

Group A: The study included 40 male patients in the case study group who were obese (body mass index >30 kg/m2), untreated, and newly diagnosed with OSAS (AHI >5/h and ESS >10 points).

Group B: Control group (n=40). Using the proper statistical techniques, additional age-matched, healthy controls from Group B were compared to the patients in Group A. Routine tests were used to examine the following variables in control and OSAS patient groups.

Anthropometry Parameters

The same observer conducted each measurement. All individuals had a thorough clinical evaluation that covers the following:

Weight on an empty stomach: Weight should be taken in the morning on an empty stomach for each subject randomized to the trial. For this assessment, each patient stood barefoot on the scales, and their weight in kilograms was recorded (kg).

Height assessment:To measure height, each patient stood against a wall barefoot, facing forward with their feet close together and heels firmly placed against the wall. On each patient's head and on the wall, the areas were lightly outlined with a pencil. Using a measuring tape, each patient's height was measured in meters from the floor to the mark.

Assessment of body mass index (BMI): BMI is calculated by dividing the weight by the square of the height. As height is measured in meters, weight is measured in kilogram (kg); therefore, the Asian-Pacific cutoff lines for BMI were used to categorize individuals into four groups: underweight (<18.5 kg/m^2^), normal weight (18.5-22.9 kg/m^2^), overweight (23-24.9 kg/m^2^), and obese (≥25 kg/m^2^) [13]. Underweight (<18.5 kg/m^2^), normal weight (18.5-24.9 kg/m^2^), overweight (25-29.9 kg/m^2^), and obese (≥30 kg/m^2^) are the four categories according to the original WHO categorization [14].

Assessment of obesity:In addition to the BMI, a number of other metrics, including waist circumference (WC), hip circumference (HC), waist/hip ratio (WHR), and neck circumference (NC) were measured for better representation of obesity in various groups [15]. 

Neck circumference assessment (NC): With the subject upright, the circumference of the neck was measured immediately below the thyroid cartilage (below Adam's apple in men) in the front and at the level of the mid-cervical spine in the rear using a flexible tape and expressed in cm [16]. The ideal cutoff value for diagnosing individuals with central obesity was found to be NC>34 cm [17].

Waist circumference (WC) assessment: To measure WC, the patient should stand on the floor, without pinching the skin. It was measured horizontally between the lower rib border and the iliac crest, mid-waist, when a regular expiration was completed and expressed in cm. Male participants in the study were considered to be obese if they had a waist circumference of more than 78 cm. The safe waist circumference limit for men is 90 cm [18].

Hip circumference (HC) assessment:The measurement utilized for the hip circumference was centimeter "cm," and it was measured as the individuals stood with their heels together at the level of maximal girth [19].

Waist-hip ratio (WHR) assessment: For the patients in our study, a waist-to-hip ratio of >0.95 in men was employed as a marker for obesity [20].

Skinfold thickness:The mid-thigh, mid-calf, and abdominal skinfold thicknesses were also assessed. Caliper measurements of skinfold thickness were made on the biceps, triceps, subscapularis, and belly [21,22].

Assessment of bicep thickness: At the front of the upper right arm, the thickness of the biceps was measured represented in mm.

Assessment of triceps thickness:* *The back of the upper right arm is where the triceps thickness was measured. A value that was used to calculate body fat was recorded on the right arm. On the back of the right triceps, the midpoint along the midline between the top of the shoulder and the bottom of the elbow was located. The skin should be pinched in such a way that the fold runs vertically. It was measured using calipers for skinfolds. The thickness of the triceps was measured as the individual stood in a straight position with hands suspended freely. The skin fold was measured for four seconds by dragging the muscle using a caliper and represented in mm. The typical thickness for men is 12 mm, whereas the normal thickness for females is 23 mm. During the measurements, the patients were advised to breathe normally and gently exhale.

Subscapular and subiliac thickness assessment: An evaluation of subscapular thickness is performed laterally and under the left shoulder blade. Just above the left iliac crest, a subiliac thickness examination is conducted.

The study adhered to the ethical standards defined by the Helsinki Declaration and the Central Ethics Committee on Human Research (CECHR), ICMR-2000 for biomedical research involving human beings. Only after written consent was obtained both in Hindi and English were the participants included in our study.

## Results

Distribution of individuals according to mean age

**Figure 1:**
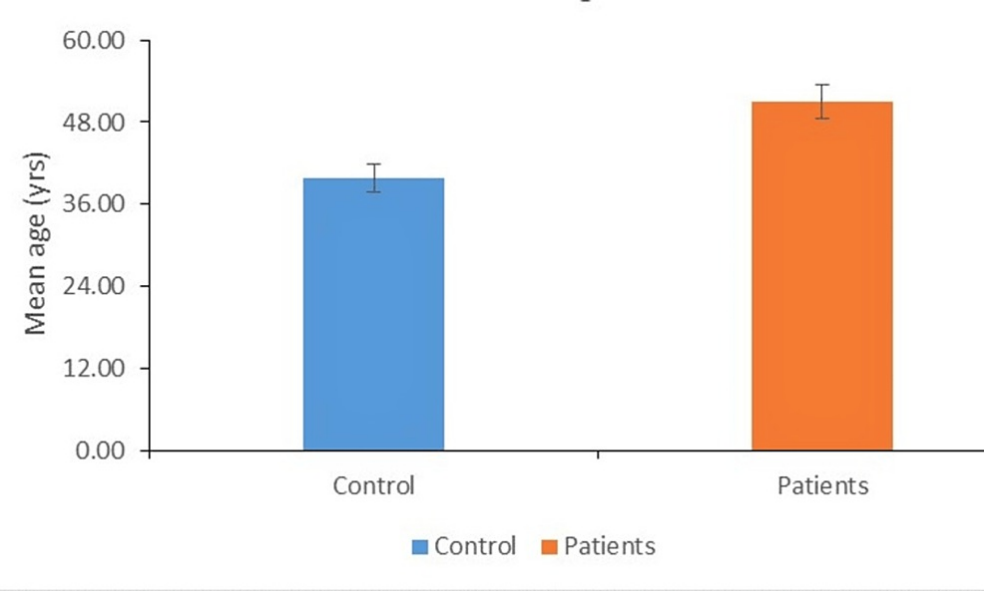
Distribution of individuals according to mean age

The distribution of subjects by mean age in the control and experimental groups is shown (Figure 1). In our study, we found that the difference between the mean age of cases and control was statistically significant (50.90±8.15 vs. 39.26±8.36; P<0.01).

Distribution of individuals according to mean weight

**Figure 2:**
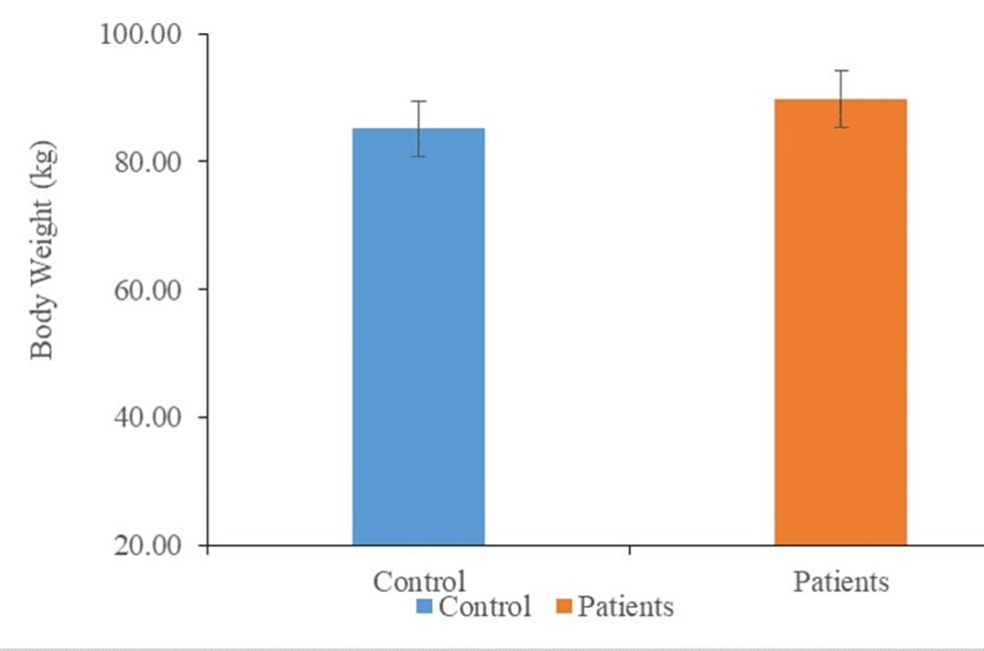
Distribution of individuals according to mean weight

The distribution of subjects in the control and experimental groups according to their mean weight is shown (Figure 2). According to the results of our investigation, the mean weight of the cases was slightly greater than the mean weight of the control (91.58±12.97 vs. 86.07±7.97; P<0.062).

Distribution of individuals according to mean height, BMI, mean neck, and waist circumference

**Figure 3:**
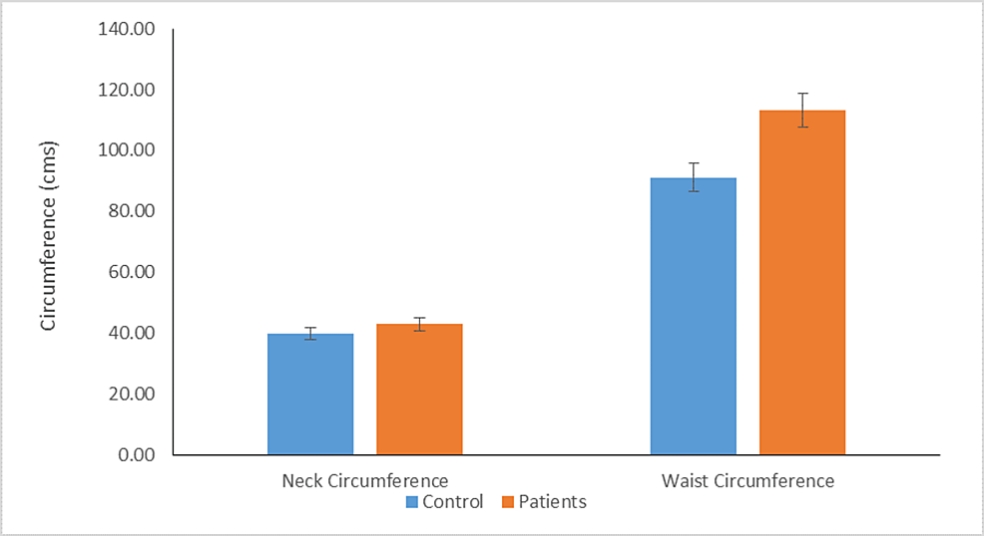
Distribution of individuals according to mean neck and waist circumference (P value <0.01)

The average height of the patient was similar to the average height of the control (167.33±8.00 vs. 166.32±5.96; P<0.513). The mean BMI of patients and controls were similar (32.67±3.27 vs. 31.14±2.01; P<0.061). The mean neck circumference of patients and control were similar (43.05±2.43 vs. 40.09±2.51). Additionally, the difference between the mean waist circumference of patients and controls was statistically significant (114.00±7.89 vs 93.10±8.20; P<0.01) (Figure 3).

Distribution of individuals according to mean waist/hip ratio

**Figure 4:**
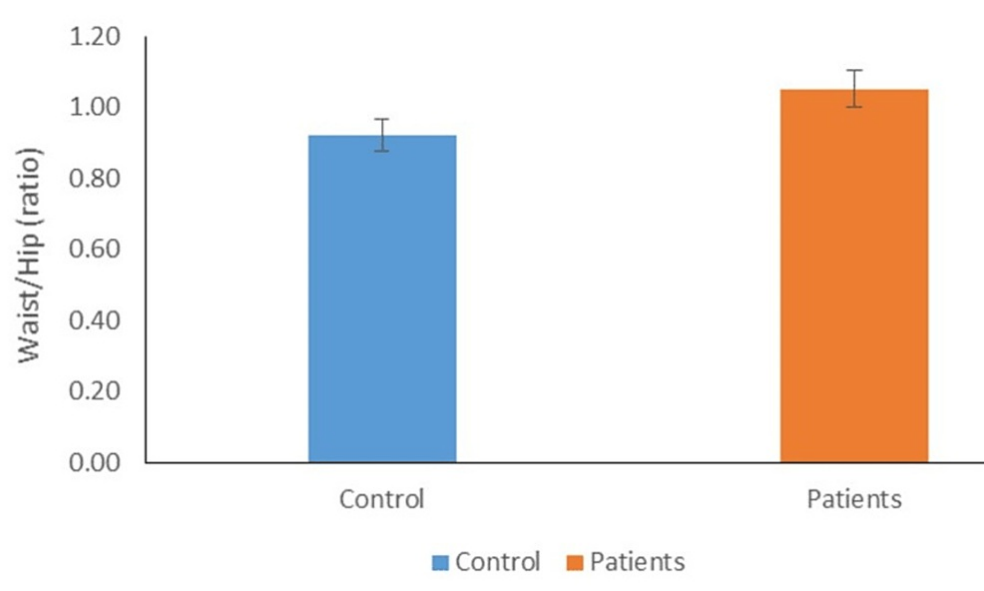
Distribution of individuals according to mean waist/hip ratio

According to their mean waist/hip ratios, the distribution of people in the control and experimental groups is shown (Figure 4). In our study, we found that patients' mean waist/hip ratios were substantially higher than those of the controls, but not statistically significant (1.09±0.25 vs. 0.96±0.19).

Distribution of individuals according to mean bicep, triceps, subscapular, and suprailiac skinfold thickness

**Figure 5:**
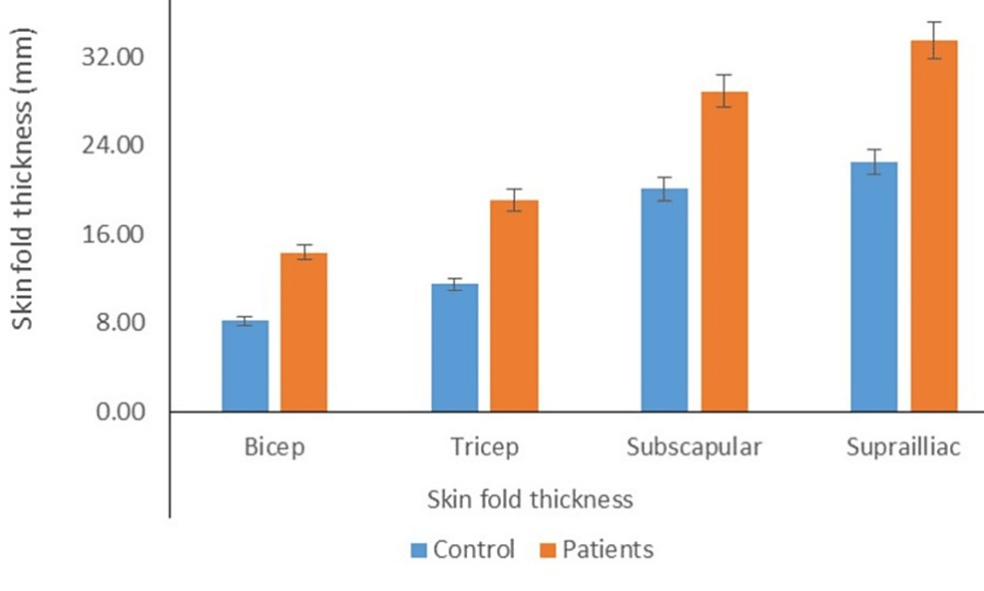
Distribution of individuals according to mean bicep, tricep, subscapular, and suprailiac skinfold thickness (P value <0.05)

The mean skinfold thickness of the biceps in the cases was substantially greater than that in controls (15.24±4.21 vs. 8.76±3.21; P<0.05). The mean skinfold thickness of the triceps in the cases was larger than the mean skinfold thickness in controls (19.56±5.48 vs. 12.14±5.44; P<0.05). Moreover, the mean suprailiac skinfold thickness (34.38±9.50 vs. 24.00±8.97; P<0.05) and mean subscapular skinfold thickness (30.19±8.26 vs. 20.74±5.85; P<0.05) of patients were considerably larger than the respective mean values for controls (Figure 5).

Distribution of individuals according to mean visceral fat and normal fat percentage

**Figure 6:**
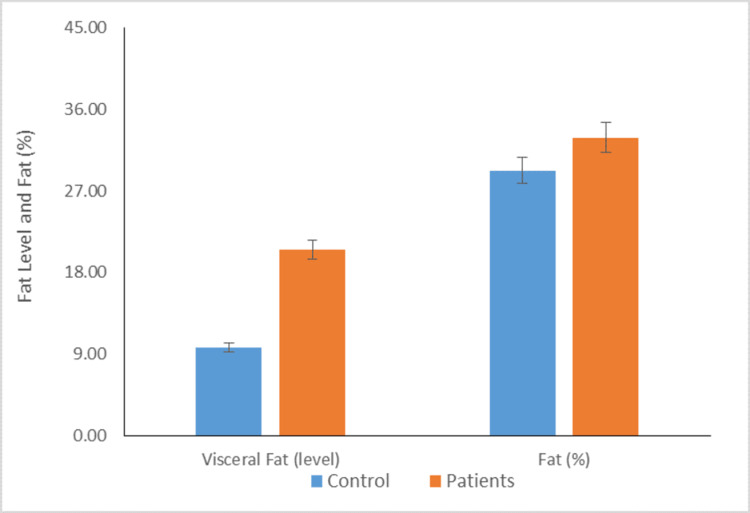
Distribution of individuals according to mean visceral fat and fat % (P value <0.01)

Figure 6 depicts that the mean visceral fat level of cases was significantly higher than the mean visceral fat level of controls (21.18±4.62 vs 10.57±4.91; P <0.01). Moreover, the normal fat % of cases was comparable to the mean fat % of control, but the results were not statistically significant (33.45±5.61 vs 29.53±10.13).

Distribution of individuals according to mean fat in different body parts

**Figure 7:**
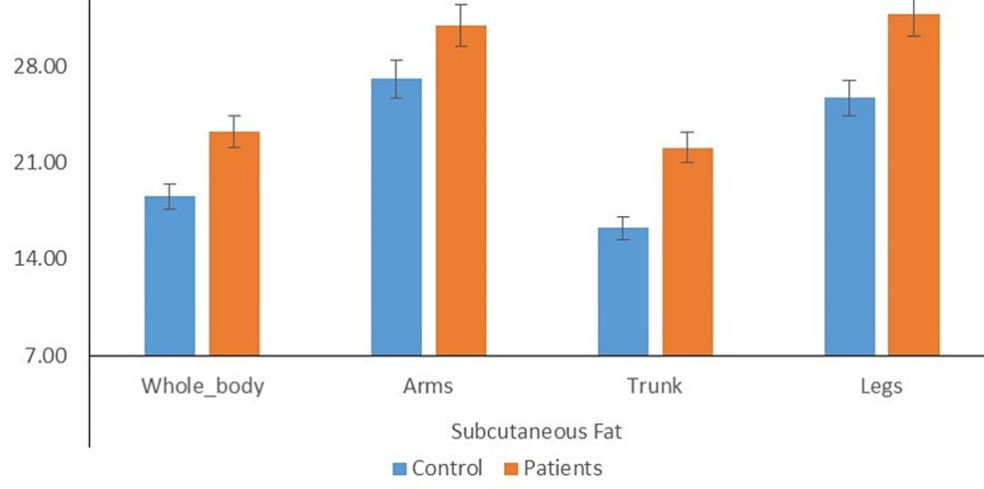
Distribution of individuals according to mean fat in different body parts (P value <0.05)

Figure 7 depicts that the mean subcutaneous whole body fat of cases was significantly higher than the mean subcutaneous whole body fat of controls (23.66±1.24 vs 19.02±1.18; P<0.01). Moreover, the mean subcutaneous arms fat of cases was comparable to the mean subcutaneous arms fat of control (31.14±1.44 vs 27.51±2.98). Furthermore, the mean subcutaneous trunk fat (22.43±2.69 vs 16.80±3.22; P<0.05) and mean subcutaneous legs fat (32.09±1.44 vs 26.13±2.64; P<0.05) of cases was significantly higher than mean subcutaneous trunk fat and subcutaneous legs fat of controls.

Distribution of individuals according to mean % of skeletal muscle in different body parts

**Figure 8:**
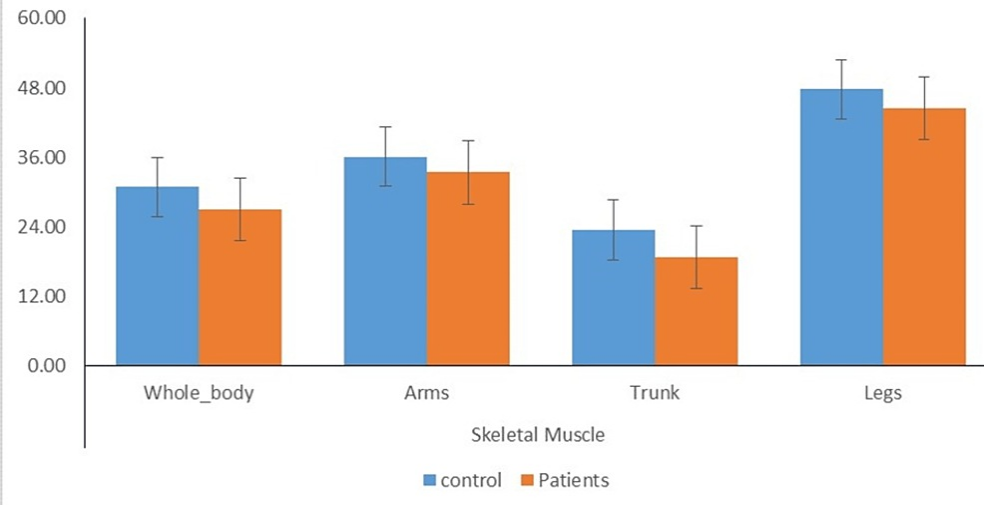
Distribution of individuals according to mean % of skeletal muscle in different body parts

Figure 8 depicts that the mean skeletal muscle whole body of the cases was comparatively lower than the mean skeletal muscle whole body of the controls (26.99±1.34 vs 30.72±1.40). Moreover, the mean skeletal muscle arms of cases were also comparatively lower than the mean skeletal muscle arms of controls (33.41±1.83 vs 35.85±3.88). Further, the mean skeletal muscle trunk (18.76±2.88vs 23.53±3.46) was comparable, and the mean skeletal muscle legs (44.56±2.20 vs 46.96±5.00) of cases was comparably lower than the mean skeletal muscle trunk of controls.

## Discussion

OSAS was defined as the recurrent collapse of the whole or partial pharynx in patients during the sleep stage [23]. Samy et al. reported that obesity is a major global health concern impacting a large portion of the population, and it is considered the main cause of OSA [24]. BMI has been traditionally used as a measure of obesity severity. However, BMI may not accurately detect adiposity in patients under the age of 30 with BMIs of less than 30 kg/m^2^ [25-27]. Neto et al. employed the Epworth sleepiness scale (ESS) and the Berlin questionnaire (BQ) to assess the prevalence and risk of OSA in obese patients undergoing bariatric surgery [28]. The relationship between the severity of OSAS and obesity demonstrates joint causality. Therefore, patients with a BMI greater than 30 were screened for obesity in the current study. To assess the relationship between OSAS and obesity, the current study measured the association between specific biochemical markers and clinical characteristics relevant to OSAS, such as BMI, skinfold caliper, NC, WC, and WHR.

In the present study, no significant difference was found in obesity-related factors (i.e., age, weight, height, WHR, and BMI) between the patient and control groups. However, previous studies have shown that the BMI and age of patients with severe OSA were higher than those with mild to moderate OSA in older populations. Additionally, OSA severity was positively and strongly associated with BMI and WHR in both sexes. Previous studies have suggested that BMI and WHR are more significant risk factors for OSA exacerbation in men. According to Himanshu et al., approximately 30% of individuals with a BMI greater than 30 kg/m2 and 50% of individuals with a BMI greater than 40 kg/m^2^ had OSAS [29].

The present study found a significant difference in measurements of obesity between the patient and control groups, specifically in circumference (waist) and skinfold thickness (bicep, tricep, subscapular, and suprailiac). The strong association between OSA and visceral obesity is well-established, but the relationship between OSA and visceral fat area remains unknown. Previous research has shown a connection between OSA and visceral obesity [30,31]. The results of this study indicate that the mean level of visceral fat in patients was significantly higher compared to the control group. No significant difference was found between the case and control groups regarding visceral fat percentage. The findings of these studies confirm previous research indicating a correlation between OSA and visceral adiposity. Moreover, independent relationships were found between AHI and visceral fat. Our study observed that the mean subcutaneous fat levels of the cases were significantly higher than those of the control group in various areas, such as the subcutaneous whole body, arms, trunk, and legs. Previous research has shown that OSA patients have higher levels of adipose tissue in their arms, trunk, and head, while the amount of adipose tissue in their legs is similar to non-OSA patients, which presents a unique pattern of adiposity [32]. Moreover, the study found no significant difference in the mean skeletal muscle mass between the control group and the patient group, including the total body, arms, trunk, and legs. However, previous studies have shown that chronic hypoxia can impact the organization of skeletal muscle, including changes in fiber size and type, and can also affect the activity levels of multiple bioenergetic enzymes [33].

The limitations of our study are less sample size and potential selection bias while recruiting study participants. Measurement errors in anthropometric assessments and the omission of confounding variables like diet and physical activity introduce potential sources of bias.

## Conclusions

Our study reported that the majority of OSAS patients’ symptoms were due to obesity and diabetes-associated conditions. Consequently, addressing altered levels of obesity-linked anthropometric variables through pharmacological interventions might hold promise as a pivotal strategy for improving symptoms associated with OSA.
